# Antidepressant mechanism of traditional Chinese medicine: Involving regulation of circadian clock genes

**DOI:** 10.1097/MD.0000000000036266

**Published:** 2023-02-02

**Authors:** Shimeng Lv, Yufei Huang, Yuexiang Ma, Jing Teng

**Affiliations:** a Department of First Clinical Medical College, Shandong University of Traditional Chinese Medicine, Jinan, China; b Ruijin Hospital Affiliated to Shanghai Jiaotong University School of Medicine, Shanghai, China; c College of Traditional Chinese Medicine, Shandong University of Traditional Chinese Medicine, Jinan, China.

**Keywords:** circadian clock genes, circadian rhythm, depression, research status, traditional Chinese medicine

## Abstract

Numerous studies have demonstrated an intimate relationship between circadian rhythm disorders and the development and prevention of depression. The biological clock genes, which constitute the molecular basis of endogenous circadian rhythms, hold promising prospects for depression treatment. Based on an extensive review of recent domestic and international research, this article presents a comprehensive analysis of how traditional Chinese medicine (TCM) intervenes in depression by regulating circadian rhythms. The findings indicate that TCM exerts its antidepressant effects by targeting specific biological clock genes such as Bmal1, clock, Arntl, Per1, Per2, Per3, Nr1d1, Cry2, and Dbp, as well as regulating circadian rhythms of hormone secretion. However, most current research is still confined to basic experimental studies, lacking clinical double-blind control trials to further validate these viewpoints. Furthermore, there is insufficient research on the signal transduction pathway between biological clock genes and pathological changes in depression. Additionally, further clarification is needed regarding the specific targets of TCM on the biological clock genes.

## 1. Introduction

Depression is a chronic, recurrent, and potentially life-threatening mental disorder. According to the World Health Organization (WHO), depression is the leading cause of disability globally and significantly diminishes the quality of life for affected individuals of all ages. Moreover, it imposes a heavy burden on both families and society. Currently, over 350 million people worldwide have been suffering from depression.^[1]^ In clinical settings, selective serotonin reuptake inhibitors constitute the primary treatment approach in Western medicine. However, most of these medications have inherent issues such as delayed efficacy and high nonresponse rates.^[2,3]^ Furthermore, they often lead to serious adverse reactions including nausea, headache, chronic sexual dysfunction, and weight gain.^[1]^ Traditional Chinese medicine (TCM) offers promising alternatives due to its characteristic features of multi-component, multi-target, and multichannel effects. Several TCM formulas have demonstrated significant efficacy with low toxicity and minimal side effects in treating depression, showcasing their potential in effectively addressing this condition.^[4]^

The circadian system consists of a central oscillator located in the hypothalamic suprachiasmatic nucleus (SCN) and a peripheral oscillator found in various peripheral tissues like the liver, lung, and skeletal muscle. Through direct light response from the retina, the SCN synchronizes the body’s internal 24-hour rhythm with the external environment. This synchronization occurs through periodic interactions between circadian clock genes, clock-controlled genes, behavioral information, and the neuroendocrine system, enabling the transmission of timing signals to peripheral oscillators. Consequently, molecular rhythms within bodily organs and cells are regulated accordingly.^[5]^ Disturbances in the circadian rhythm can increase the susceptibility of patients to depression. Notably, clinical data surveys have revealed that 20% to 30% of depressed patients experience circadian rhythm disorders.^[6]^ Sleep disorder represents one of the most prominent clinical manifestations of circadian rhythm disruptions in depressed individuals, with up to 90% of patients exhibiting varying degrees of sleep-related symptoms.^[7]^ By regulating the expression of circadian clock genes, the improvement of circadian disorders has emerged as a significant target for depression intervention. Moreover, a disrupted sleep cycle in depressed patients, when effectively addressed, contributes to the restoration of normal circadian rhythms. Additional research has identified the restoration of rhythmical expression of circadian clock genes involved in regulating circadian rhythms.^[8,9]^

This review was aimed to summarize recent relevant studies conducted abroad, explore the pathological relationship and regulatory mechanisms between the circadian clock system and depression, and assess the mechanism by which TCM interventions based on the regulation of circadian rhythm offer therapeutic benefits for depression, thereby providing valuable references for future basic research and clinical applications.

## 2. The relationship between circadian rhythm and depression

Due to the molecular basis of endogenous circadian rhythm, external light is transmitted to the SCN through the retino-hypothalamic bundle, forming a series of transcriptional and translational feedback loops. These loops synchronously regulate the expression of peripheral circadian clock genes, ensuring the body synchronized with the external circadian alternation. The core transcriptional translation feedback loop consists mainly of the transcription factors brain and muscle ARNT-like 1 (Bmal1)/circadian locomotor output cycles kaput (clock), as well as cryptochromes (Crys). Bmal1and clock act as positive regulators of the circadian clock. Upon forming heterodimers, they bind to E-box and activate the transcription of target genes such as period [Pers] and Crys. Simultaneously, an additional feedback loop involving nuclear receptors (nuclear receptor subfamily 1 group D member and retinoic acid receptor-related orphan receptors [Rorα]) also regulates circadian rhythm through E-box, controlling the expression of Bmal1via rev response elements. Together, these mechanisms drive the circadian expression program of circadian clock genes.^[10]^

In the detection of clinical samples, abnormal expression of Bmal1, Per1-3, and nuclear receptor subfamily 1 group D member (α) involved in regulating circadian rhythm was found in patients with depression.^[11]^ Similarly, depression model rats displayed abnormal expression of biological clock genes, resulting in disrupted circadian oscillations in the basal lateral amygdala for clock, Cry2, Per1, Per3, Id2, Rev-erbα, Ror-β, and Ror-γ.^[12,13]^ Moreover, there are reports highlighting the crucial role played by SCN and the clock gene in the hippocampus during the pathological progression of depression.^[14]^ In a mouse model of circadian rhythm disorder induced by abnormal light, depressive behavior was found to be associated with impaired expression levels of Per1 and Per2 genes.^[15]^ Additionally, a decrease in Per2 expression in the lateral habenular nucleus may be linked to an increase in depressive behavior.^[16]^ Importantly, the presence of Per1 gene polymorphism and changes in the microstructure of white matter in emotional brain regions can serve as indicators for predicting the risk of depression in early-stage patients.^[17]^

## 3. Biological clock genes regulate the physiological and pathological mechanisms of depression

In the basic research model of circadian rhythm disorder, Hou et al discovered that rats exhibited depression-like behavior. Their mechanism study revealed a decrease in the number of neurons and astrocytes in the prefrontal cortex, along with damaged synapses of pyramidal cells.^[18]^ The neurotransmitter 5-HT belongs to the category of monoamine neurotransmitters, which play a role in regulating emotional cognition and sleep. According to the classic monoamine hypothesis, depression may be caused by the depletion of monoamine neurotransmitter levels in the central nervous system.^[1]^ Evidence indicates that biological clock genes are involved in the physiological and pathological processes of depression through the 5-HT system. Additionally, the use of antidepressants that regulate neurotransmitters involves the regulation of circadian rhythms.^[19]^ It was also found that chronic unpredictable stress (CUMS) causes depressive-like behavior in rats by acting on clock genes leading to phase advancement of glutamate (Glu) and γ-aminobutyric acid.^[20]^ Besides, it was also found that CUMS causes depressive-like behavior in rats by acting on clock genes leading to phase advancement of Glu and γ-aminobutyric acid.^[20]^ Otsuka et al demonstrated that the Rev-erbα gene can regulate negative emotions and behaviors in mice by acting on the 5-hydroxytryptaminergic system.^[21]^ The hypothalamic–pituitary–adrenal (HPA) axis is a significant part of the neuroendocrine system, which is involved in controlling the response to stress. When the HPA axis is activated, the paraventricular nucleus of the hypothalamus releases corticotropin-releasing hormone, thereby signaling the anterior pituitary gland to secrete adrenocorticotropic hormone into the blood stream, which in turn acts on the adrenal cortex to stimulate the secretion of cortisol (CORT).^[22]^ Hyperactivity of the HPA axis is a pivotal factor in the development of depression pathology,^[23]^ which may be the potential mechanism by which the deletion of the Per2 gene causes depressive-like behavior in mice.^[24]^ Depression like behavior was also observed in monkeys with Bmal1 gene knockout, and an increase in blood CORT levels was further revealed.^[25]^ A follow-up study found that SCN-Bmal1-KD mice derived from inhibition of the Bmal1 gene table in mouse SCN neurons using shRNA could serve for a new depression model.^[26]^

**Figure 1.**
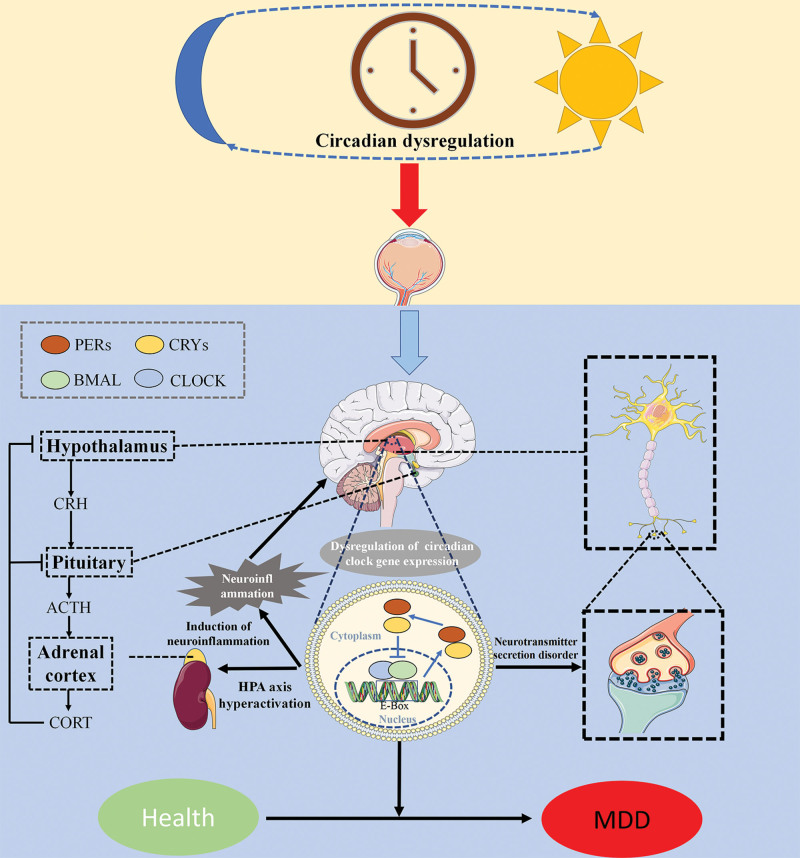
Mechanism of biological clock genes inducing depression. ACTH = Adrenocorticotropic hormone; Bmal1 = brain and muscle ARNT-like 1; Clock = circadian locomotor output cycles protein kaput; CORT = cortisol; CRH = corticotropin-releasing hormone; Cry = cryptochrome; HPA = hypothalamic–pituitary–adrenal; MDD = major depressive disorder; Per = period.

Abundant evidence supports the role of neuroinflammation in inducing the development of depression pathology. Inflammation increases patients’ susceptibility to depressed mood, and elevated pro-inflammatory markers and the use of pro-inflammatory medications in depressed patients elevate the risk of developing depression.^[27]^ Chen et al discovered that the Per2 gene alleviates neuroinflammation-induced depressive-like behaviors by regulating the rhythm-control gene chemokine Rantes.^[28]^ Sleep deprivation given to rats disrupts their circadian rhythm and triggers neuroinflammation, resulting in depressive symptoms.^[29]^ Figure 1 illustrates the mechanism of action of circadian rhythm disruption in inducing depression.

## 4. TCM Regulating the circadian rhythm in the treatment of depression

### 4.1. TCM compounds

Modified *Sinisan* is derived from Zhang Zhongjing “*Treatise on Febrile Diseases*” through the addition and subtraction of ingredients. There is evidence suggesting that Modified *Sinisan* can effectively treat depression in animal models induced by CUMS by regulating hormone secretion and adjusting their rhythm.^[30]^ A subsequent study revealed that Modified *Sinisan* achieved its antidepressant effect primarily by modulating the content and temporal rhythm of Per, Cry, Bmal1, and clock genes associated with the SCN in the hypothalamus.^[31,32]^ *YishenNingxin* has been found to improve symptoms of insomnia and accompanying depressive states by modulating the concentration levels of salivary MT, CORT, 5-HT, and circadian rhythm disruption in patients.^[33]^ *Lily Bulb and Rehmannia Decoction*, a formula for lily disease treatment, was first published in “*The Essentials of the Golden Poverty*” and consists of 7 fresh lilies and 1 L of raw dihuang juice.^[34]^ Zhang et al demonstrated that *Lily Bulb and Rehmannia Decoction* improved depression-like symptoms in rats and RNA sequencing indicated the involvement of the circadian rhythm gene Nr1d1 in this regulation.^[35]^ Additionally, it has been reported that the *Chaihu-Baishao* hair pair can exert antidepressant effects by regulating the Per2 and Cry2 genes.^[36]^

### 4.2. Active ingredients in TCM

Beta-asarone is identified as the primary active ingredient in *Acorus tatarinowii*. It exhibits pharmacological effects such as inhibiting inflammatory reactions, reducing oxidative stress, improving energy metabolism, decreasing cell excitatory toxicity, and protecting the blood-brain barrier.^[37]^ Dong et al found that β-asarone improved the depressive state of rats by upregulating the expression of the circadian gene Per1 in the brain region associated with depression.^[38]^ *Poria cocos*, characterized by a mild nature and a sweet and light taste, has diuretic properties and is beneficial for nourishing the spleen and stomach, as well as calming the heart and mind. *Poria polaris* extract was found to ameliorate neurotransmitter and circadian rhythm disorders in CUMS rats by regulating circadian genes Arntl, Per1, Per2, Per3, and Nr1d1, leading to improved expression levels.^[39]^ Paeoniflorin, a monoterpenoid glycoside compound derived from the roots of *Paeonia lactiflora*, exhibits antioxidant, anti-inflammatory, anti-fibrotic, and neuroprotective effects.^[40]^ Studies have shown that depression model rats exhibit disrupted rhythms of clock gene expression in the hippocampus and administration of paeoniflorin significantly improves the changes in clock and Bmal1 gene expression rhythms, thereby exerting an antidepressant effect.^[41]^ Theanine, the primary amino acid in tea, especially green tea, contributes to its sweet and fresh flavor. Theanine has been found to regulate the circadian rhythm of HPA axis secretion, thus demonstrating its ameliorative effect on depression.^[42]^

### 4.3. TCM therapy

Acupuncture, an integral part of Chinese medicine, has been demonstrated to have favorable antidepressant effects, either alone or in combination with other therapies.^[43]^ Hong et al discovered that acupuncture modulates blood glucose levels, depressed mood, and CORT circadian rhythm in rats with diabetic depression models. This mechanism may be achieved by down-regulating the expression of the Per2 gene within the SCN of diabetic depressed rats, thus reducing the feedback inhibition of positive processes.^[44]^ Electroacupuncture, a modified acupuncture therapy combined with electrical stimulation, has been widely utilized in neurological disorders such as stroke, depression, insomnia, and stress-related disorders. It has proven effective in the clinical treatment of depression.^[45]^ In a chronic stress-induced depression model, rats exhibited disrupted body temperature and melatonin circadian rhythms, while administration of electroacupuncture to Yintang and Baihui points alleviated these pathologies and improved depressive-like behavior.^[46]^ Early life experiences play a crucial role in the development of human brain structure and function. Adverse events during childhood can impact an individual, leading to behavioral and neurological changes in adulthood, which is considered a risk factor for depression.^[47]^ Zheng et al administered electroacupuncture to the Baihui and Yintang points in a mother–infant separation-induced depression model and observed improvements in the depression-like phenotype of the animals. RNA sequencing revealed that the regulation of biological clock genes Per2 and Dbp was involved in this process.^[48]^The mechanism of action of TCM is shown in Table 1.

**Table 1:** Mechanisms of action for traditional Chinese medicine.

Intervention	Molding method	Experimental subjects	Dosage or intervention points	Mechanism involved	References
Modified Sinsan	CUMS	SPF male SD rats	1.69 g/mL	Regulating hormone secretion and regulating its rhythm	^[30]^
Modified Sinsan	CUMS	SPF male SD rats	16.9 g/kg	Regulating the expression levels of Per and Cry at time points in SCN and altering their time rhythm	^[31]^
Modified Sinisan	CUMS	SPF male SD rats	16.9 g/kg	Regulating the expression levels of Bmal1 and Clock related time points in SCN and altering their time rhythm	^[32]^
Lily Bulb and Rehmannia Decoction	CUMS	Male C57BL/6J juvenile mice	150 g/kg	Regulation of circadian rhythm gene Nr1d1 expression	^[35]^
Chaihu-Baishao hair pair	CUMS	SPF male SD rats	4.0176, 2.0088, 1.0044 g/kg	Regulating the expression of circadian rhythm genes Per2 and Cry2	^[36]^
β-Asarone	CUMS	SPF male SD rats	12.5, 25 g/kg	Regulation of the expression of circadian rhythm gene Per1 in the brain region of	^[38]^
Poria polaris extract	CUMS	SPF male SD rats	55.45 mg/mL	Regulation of the expression levels of circadian rhythm genes Arntl, Per1, Per2, Per3, Nr1d1	^[39]^
Paeoniflorin	CUMS	SPF male SD rats	5, 15 mg/kg	Improving the Rhythmic Changes of Clock and Bmal1 Expression in Biological Clock Genes	^[41]^
Theanine	Residential invasion	Male ddY mice	5–100 μg/mL	Regulating the circadian rhythm secretion of the HPA axis	^[42]^
Acupuncture	STZ + High fat and high sugar diet	SPF male SD rats	Housanli, Sanyinjiao, and Baihui	Downregulation of Per2 gene expression in SCN	^[44]^
Electroacupuncture	CUMS	SPF male SD rats	Baihui and Yintang	Regulating body temperature and melatonin circadian rhythm changes	^[46]^
Electroacupuncture	Maternal separation	Male and female Wister rats	Baihui and Yintang	Regulating biological clock genes Per2 and Dbp	^[48]^

Arntl = Aryl hydrocarbon receptor nuclear translocator-like, Bmal1 = Brain and muscle ARNT-like 1, Clock = circadian locomotor output cycles protein kaput, Cry = Cryptochrome, CUMS = Chronic unpredictable mild stress, Dbp = Albumin promoter D-site binding protein, HPA = hypothalamic-pituitary-adrenal, Nr1d1 = Nuclear receptor subfamily 1 group D member 1, Per = Period, SCN = suprachiasmatic nucleus, STZ = streptozocin.

## 5. Discussion

Depression is a prevalent mood disorder characterized by persistent feelings of sadness, decreased interest, and reduced energy. It is a chronic, recurrent, and potentially life-threatening mental disorder.^[1]^ The circadian rhythm refers to the regular oscillating phenomenon with a roughly 24-hour cycle, which manifests in various physiological, biochemical, and behavioral activities of an organism. This rhythm is driven by the periodicity of biological clock genes and clock-controlled genes.^[49]^ In recent years, research focusing on the pathogenesis of depression has increasingly emphasized the hypothesis of circadian rhythm disorder.^[50]^ Studies have revealed that dysregulation of biological clock genes contributes to the development of depression through the induction of neuroinflammation, abnormal activity of the HPA axis, and disturbances in neurotransmitter secretion. Consequently, TCM can exhibit antidepressant effects by targeting specific biological clock genes such as Cry, Bmal1, clock, Arntl, Per1, Per2, Per3, Nr1d1, and Cry2, while also regulating circadian rhythms associated with body temperature and hormone secretion. However, current research primarily relies on animal models, lacking sufficient clinical validation to evaluate therapeutic effects on patients. Furthermore, although the effective ingredients of TCM have demonstrated certain therapeutic effects, stability, solubility, and permeability through the blood-brain barrier remain problematic, significantly limiting their efficacy. Mechanism research on TCM primarily focuses on identifying targets among biological clock genes, with inadequate exploration of the upstream and downstream pathways associated with these genes and depression pathology. Additionally, further clarification is needed regarding the specificity of TCM and its interaction with biological clock gene targets.

In future investigations, it is imperative to conduct a considerable number of clinical randomized double-blind controlled trials guided by TCM theory to explore whether TCM can effectively treat depression by regulating the patient’s biological rhythm. Consequently, researching on targeted delivery systems for TCM becomes crucial in order to increase drug concentration and duration of action within the central nervous system, thus improving therapeutic outcomes. Additionally, incorporating reverse validation methods such as agonists and blockers will help elucidate specific biological clock gene targets affected by TCM. Simultaneously, leveraging multi-omics technology will enrich our understanding of how TCM regulates biological clock genes and further enhance our comprehension of its overall intervention mechanism providing guidance for subsequent basic research and clinical applications.

## Author contributions

**Writing – original draft:** Shimeng Lv.

**Writing – review & editing:** Yufei Huang, Yuexiang Ma, Jing Teng.

## References

[1] Lv S Zhao Y Wang L. Antidepressant active components of bupleurum chinense dc-paeonia lactiflora pall herb pair: pharmacological mechanisms. Biomed Res Int. 2022;2022:1024693. 36408279 10.1155/2022/1024693 PMC9668458

[2] Qu SY Li XY Heng X. Analysis of antidepressant activity of huang-lian jie-du decoction through network pharmacology and metabolomics. Front Pharmacol. 2021;12:619288. 33746756 10.3389/fphar.2021.619288 PMC7970346

[3] Wei Y Chang L Hashimoto K. Molecular mechanisms underlying the antidepressant actions of arketamine: beyond the NMDA receptor. Mol Psychiatry. 2022;27:559–73. 33963284 10.1038/s41380-021-01121-1 PMC8960399

[4] Chi X Wang S Baloch Z. Research progress on classical traditional Chinese medicine formula Lily Bulb and Rehmannia Decoction in the treatment of depression. Biomed Pharmacother. 2019;112:108616. 30780102 10.1016/j.biopha.2019.108616

[5] Han Z Shen Y Xun Y. Research progress on the relationship between circadian rhythm disorder and non-alcoholic fatty liver disease. J Integr Tradit Chinese Western Med Hepatol. 2022;32:1150–2.

[6] Sato S Bunney B Mendoza-Viveros L. Rapid-acting antidepressants and the circadian clock. Neuropsychopharmacology. 2022;47:805–16. 34837078 10.1038/s41386-021-01241-w PMC8626287

[7] Tao S Chattun MR Yan R. TPH-2 gene polymorphism in major depressive disorder patients with early-wakening symptom. Front Neurosci. 2018;12:827. 30519155 10.3389/fnins.2018.00827 PMC6251472

[8] Hasler BP Buysse DJ Kupfer DJ. Phase relationships between core body temperature, melatonin, and sleep are associated with depression severity: further evidence for circadian misalignment in non-seasonal depression. Psychiatry Res. 2010;178:205–7. 20471106 10.1016/j.psychres.2010.04.027 PMC2914120

[9] Chen SJ Deng YT Li YZ. Association of circadian rhythms with brain disorder incidents: a prospective cohort study of 72242 participants. Transl Psychiatry. 2022;12:514. 36517471 10.1038/s41398-022-02278-1 PMC9751105

[10] Wang S Liu X Liu L. Research progress on the regulation of biological clock genes by traditional Chinese medicine in the prevention and treatment of insomnia. World J Tradit Chinese Med. 2023;18:734–8.

[11] Li JZ Bunney BG Meng F. Circadian patterns of gene expression in the human brain and disruption in major depressive disorder. Proc Natl Acad Sci U S A. 2013;110:9950–5. 23671070 10.1073/pnas.1305814110 PMC3683716

[12] Christiansen SL Bouzinova EV Fahrenkrug J. Altered expression pattern of clock genes in a rat model of depression. Int J Neuropsychopharmacol. 2016;19:pyw061. 27365111 10.1093/ijnp/pyw061 PMC5137278

[13] Savalli G Diao W Schulz S. Diurnal oscillation of amygdala clock gene expression and loss of synchrony in a mouse model of depression. Int J Neuropsychopharmacol. 2014;18:pyu095. 25522426 10.1093/ijnp/pyu095 PMC4376549

[14] Jiang WG Li SX Liu JF. Hippocampal CLOCK protein participates in the persistence of depressive-like behavior induced by chronic unpredictable stress. Psychopharmacology (Berl). 2013;227:79–92. 23263459 10.1007/s00213-012-2941-4

[15] Moriya S Tahara Y Sasaki H. Housing under abnormal light-dark cycles attenuates day/night expression rhythms of the clock genes Per1, Per2, and Bmal1 in the amygdala and hippocampus of mice. Neurosci Res. 2015;99:16–21. 26026603 10.1016/j.neures.2015.05.005

[16] Li Y Li G Li J. Depression-like behavior is associated with lower Per2 mRNA expression in the lateral habenula of rats. Genes Brain Behav. 2021;20:e12702. 32964673 10.1111/gbb.12702

[17] Zhao R Sun JB Deng H. Per1 gene polymorphisms influence the relationship between brain white matter microstructure and depression risk. Front Psychiatry. 2022;13:1022442. 36440417 10.3389/fpsyt.2022.1022442 PMC9691780

[18] Hou Y Wang Y Song S. Long-term variable photoperiod exposure impairs the mPFC and induces anxiety and depression-like behavior in male wistar rats. Exp Neurol. 2022;347:113908. 34710402 10.1016/j.expneurol.2021.113908

[19] Daut RA Fonken LK. Circadian regulation of depression: a role for serotonin. Front Neuroendocrinol. 2019;54:100746. 31002895 10.1016/j.yfrne.2019.04.003 PMC9826732

[20] Wang P Gao X Zhao F. Study of the neurotransmitter changes adjusted by circadian rhythm in depression based on liver transcriptomics and correlation analysis. ACS Chem Neurosci. 2021;12:2151–66. 34060807 10.1021/acschemneuro.1c00115

[21] Otsuka T Le HT Thein ZL. Deficiency of the circadian clock gene Rev-erbα induces mood disorder-like behaviours and dysregulation of the serotonergic system in mice. Physiol Behav. 2022;256:113960. 36115382 10.1016/j.physbeh.2022.113960

[22] Frankiensztajn LM Elliott E Koren O. The microbiota and the hypothalamus–pituitary–adrenocortical (HPA) axis, implications for anxiety and stress disorders. Curr Opin Neurobiol. 2020;62:76–82. 31972462 10.1016/j.conb.2019.12.003

[23] Wang XL Feng ST Wang YT. A neuroprotective monoterpenoid glycoside with promising anti-depressive properties. Phytomedicine. 2021;90:153669. 34334273 10.1016/j.phymed.2021.153669

[24] Russell AL Miller L Yi H. Knockout of the circadian gene, Per2, disrupts corticosterone secretion and results in depressive-like behaviors and deficits in startle responses. BMC Neurosci. 2021;22:5. 33509094 10.1186/s12868-020-00607-y PMC7841886

[25] Qiu P Jiang J Liu Z. BMAL1 knockout macaque monkeys display reduced sleep and psychiatric disorders. Natl Sci Rev. 2019;6:87–100. 34691834 10.1093/nsr/nwz002 PMC8291534

[26] Landgraf D Long JE Proulx CD. Genetic disruption of circadian rhythms in the suprachiasmatic nucleus causes helplessness, behavioral despair, and anxiety-like behavior in mice. Biol Psychiatry. 2016;80:827–35. 27113500 10.1016/j.biopsych.2016.03.1050 PMC5102810

[27] Kohler O Krogh J Mors O. Inflammation in depression and the potential for anti-inflammatory treatment. Curr Neuropharmacol. 2016;14:732–42. 27640518 10.2174/1570159X14666151208113700 PMC5050394

[28] Chen X Hu Q Zhang K. The clock-controlled chemokine contributes to neuroinflammation-induced depression. FASEB J. 2020;34:8357–66. 32329129 10.1096/fj.201900581RRR

[29] Xing C Zhou Y Xu H. Sleep disturbance induces depressive behaviors and neuroinflammation by altering the circadian oscillations of clock genes in rats. Neurosci Res. 2021;171:124–32. 33785408 10.1016/j.neures.2021.03.006

[30] Liu Y. The effect of seasoned administration of Tiaogan Formula and Sini Powder on the circadian rhythm of CMUS rats. Guangzhou Univ Tradit Chinese Med. 2010.

[31] Liu L Chen Q Liu Y. The expression of SCN biological clock genes in the hypothalamus of rats with stress induced depression and their circadian rhythm after administration of Jiawei Sini San at different times. Med Theory Pract. 2018;31:781–4.

[32] Wu L Chen Q Liu Y. Effects of timed administration of Jiawei Sini San on the expression and circadian rhythm of SCN clock genes Per and Cry in the hypothalamus of rats with stress induced depression. Pharmacol Clin Appl Tradit Chinese Med. 2014;30:92–8.

[33] Chai R. Clinical observation and related mechanism study on the treatment of insomnia of heart and spleen deficiency type with modified yishen ningxin formula. Shanghai Univ Tradit Chinese Med. 2020.

[34] Qin G Yi Q Zhang H. Research progress on the pharmacological effects of Baihe Dihuang Tang. Shandong J Tradit Chinese Med. 2023;42:299–303.

[35] Zhang H Chi X Pan W. Antidepressant mechanism of classical herbal formula lily bulb and Rehmannia decoction: insights from gene expression profile of medial prefrontal cortex of mice with stress-induced depression-like behavior. Genes Brain Behav. 2020;19:e12649. 32129566 10.1111/gbb.12649

[36] Yuqi F. Study on the antidepressant mechanism of Chaihu Baishao based on transcriptomics and network pharmacology. Heilongjiang Univ Tradit Chinese Med. 2022.

[37] Lu Z Lu Q Ding Y. Study on the protective effect and mechanism of β-Asarone on astrocyte damage induced by oxygen glucose deprivation/re oxygenation. Chongqing Med. 2023;52:161–166 + 171.

[38] Dong H Zhang C Wang J. The effect of β-Asarone on the expression of the circadian rhythm gene Per1 in depression model rats. Chinese J New Drugs. 2015;24:823–6.

[39] Meng M Feng Y Wang P. Experimental study on the regulation of neurotransmitters and circadian rhythm in CUMS rats by Poria polaris extract. Chinese Herbal Med. 2020;51:118–26.

[40] Dan M Chen Li. The effect of paeoniflorin on the expression of NLRP3 inflammasomes in early brain injury rats with subarachnoid hemorrhage. Stroke Neurological Diseases. 2023;30:154–164 + 174.

[41] Wang J. The effect and mechanism of paeoniflorin on the biological rhythm of depression model rats. Heilongjiang Univ Tradit Chinese Med. 2015.

[42] Unno K Iguchi K Tanida N. Ingestion of theanine, an amino acid in tea, suppresses psychosocial stress in mice. Exp Physiol. 2013;98:290–303. 22707502 10.1113/expphysiol.2012.065532

[43] Li Q Chen Q Yang S. Meta analysis of acupuncture treatment for postpartum depression. Chinese J Tradit Chinese Med Library Inform. 2023;47:103–9.

[44] Hong X Wang B Huang J. Effect of acupuncture on the circadian rhythm of serum CORT and the expression of Per1 and Per2 genes in SCN of diabetes depression model rats. Liaoning J Tradit Chinese Med. 2018;45:483–6.

[45] Zhao T Zhu T Zhang J. The effect of electroacupuncture on the CREB/BDNF/TrkB signaling pathway in the hippocampus of depression rats. J Hainan Med College. 2023;29:516–22.

[46] Yao H Song H Mo Y. Effect of electroacupuncture on body temperature and melatonin circadian rhythm of chronic stress induced depression model rats. China Acupuncture and Moxibustion. 2014;34:685–9. 25233660

[47] Zhou L Zuotian W Wang G. Study on the effects and related mechanisms of maternal infant separation on neural development and the occurrence and development of depression. J Clin Psychiatry. 2021;31:77–9.

[48] Zheng Y He J Guo L. Transcriptome analysis on maternal separation rats with depression-related manifestations ameliorated by electroacupuncture. Front Neurosci. 2019;13:314. 31024237 10.3389/fnins.2019.00314 PMC6460510

[49] Wang J Hou W Qin X. Research progress on circadian rhythm. Chinese J Tradit Chinese Med. 2021;46:3240–8. 10.19540/j.cnki.cjcmm.20210308.601 34396743

[50] Tao S. Association of sTPH2 gene polymorphism with circadian rhythm disorders related to sleep in depression and related resting brain functional characteristics. Nanjing Med Univ. 2019.

